# Shared Decision Making: The Prerequisite for Substantial Autonomy in Evidence‐Based Practice

**DOI:** 10.1111/jep.70171

**Published:** 2025-06-23

**Authors:** André Demambre Bacchi

**Affiliations:** ^1^ Faculty of Health Sciences Federal University of Rondonópolis Rondonópolis Mato Grosso Brazil

**Keywords:** evidence‐based medicine, medical ethics, person‐centred medicine, philosophy of medicine

Evidence‐Based Practice (EBP) has been widely accepted as the dominant paradigm in contemporary healthcare, traditionally grounded in a triad of principles: the best available scientific evidence, the clinician's expertise, and the patient's values and preferences [1]. However, a critical gap persists in the implementation of this model: the third pillar, patient values and preferences, often remains a theoretical ideal rather than a tangible component of routine care [2]. To address this disconnection, EBP requires conceptual refinement. Shared decision making (SDM) must be understood not as a discretionary tool but as the fundamental mechanism through which substantial patient autonomy is operationalized, thereby transforming the third pillar from an aspirational statement into a clinical reality [3].

Contemporary bioethics positions autonomy as a foundational principle. However, it is crucial to distinguish between *formal autonomy* ‐ the abstract legal right to choose—and *substantial autonomy*, the actual capacity to make informed, deliberate and value‐congruent decisions [4]. Although traditional informed consent satisfies the conditions of formal autonomy, it often falls short of enabling substantial autonomy. A patient may formally consent to a proposed intervention without genuinely understanding its implications or viable alternatives, ultimately making choices that do not authentically reflect their values [5].

Table 1 outlines the essential distinctions between formal and substantial autonomy within the EBP framework. Formal autonomy corresponds to the baseline recognition of patient rights, which is typically enacted through standard informed consent procedures. By contrast, substantial autonomy facilitated by SDM embodies the patient's effective ability to make informed, reflective and value‐oriented decisions.

**Table 1:** Formal autonomy versus substantial autonomy in the context of evidence‐based practice.

Aspect	Formal autonomy	Substantial autonomy
Definition	Legal and ethical right to make decisions about one's own care	Actual and effective capacity to make informed decisions aligned with personal values
Evidence‐based practice basis	Theoretical acknowledgement of the third pillar (values and preferences)	Concrete operationalization of the third pillar of EBP
Mechanism of expression	Informed consent	Shared decision making (SDM)
Nature of information	Unidirectional (clinician → patient)	Bidirectional, deliberative and collaborative
Patient involvement	Passive (receives information and signs consent)	Active (engages in value exploration, deliberation and co‐decision)
Temporality	One‐time event (e.g., prescription, signing a document)	Ongoing and iterative process
Resulting decision	Legally valid	Clinically meaningful and personally relevant
Professional's role	Neutral informant	Facilitator of value‐sensitive deliberation
Final outcome	Legal and ethical protection	Care that is genuinely patient‐centred

Within this framework, SDM emerges as the mechanism that truly operationalizes the third pillar of EBP, translating ‘values and preferences’ from abstract concepts into concrete elements of clinical decision‐making. SDM entails a structured process comprising bidirectional communication about the clinical condition, presentation of evidence‐based options, elicitation of patient values and preferences, collaborative deliberation and implementation of a consensual decision. This approach markedly transcends the conventional informed consent process, which is often reduced to unidirectional transfer of information followed by formal approval [6].

Despite the growing recognition of SDM's relevance, its implementation remains hindered by substantial challenges across multiple levels. At the individual level, barriers include limited communication skills among clinicians, reluctance to relinquish traditional medical paternalism and low health literacy among patients. At the organizational level, key obstacles include time constraints during consultations, a lack of institutional incentives and an organizational culture that undervalues patient participation. Systemically, the absence of health policies promoting SDM and curricular gaps in the training of healthcare professionals further limit its integration into practice [7].

Recognizing SDM as a cornerstone for the exercise of substantial patient autonomy within EBP carries significant implications across healthcare domains. In clinical practice, this necessitates the integration of decision support tools into routine workflows, the development of clinician competencies in value‐sensitive communication and the adoption of metrics that evaluate decision quality beyond traditional clinical outcomes [8]. In research, attention must be expanded beyond efficacy and effectiveness to include the alignment of interventions with patient values and the creation of methodologies that capture the patient's experience in the decision‐making process [9]. In the realm of health policy, structural incentives to promote SDM, the inclusion of decision quality indicators in performance metrics and curricular reforms in health professional education are imperative steps forward [10].

The effective implementation of SDM is also an ethical imperative grounded in the principle of distributive justice. While formal autonomy establishes a universal right to choose, it operates under the principle of equality, treating all individuals the same regardless of their actual capacity to exercise that right. Paradoxically, this approach may perpetuate inequities by failing to account for differential needs and capabilities. In contrast, substantial autonomy rests on the principle of equity, recognizing that individuals require differing forms and levels of support to achieve meaningful decision‐making capacity. The absence of such support disproportionately affects marginalized populations, reinforcing a social gradient of autonomy that mirrors and sustains existing health inequities.

Therefore, we propose a recalibration of the EBP model, wherein SDM is no longer treated as an ancillary methodology but rather acknowledged explicitly as the operational mechanism upon which the third pillar, patient values and preferences, depends. The concept of substantial autonomy provides a compelling theoretical framework to explain why SDM is not merely desirable, but essential in truly patient‐centred EBP. This reframing offers a pathway to reconcile the perceived tension between the standardization inherent in EBP and the personalization required for compassionate individualized care. SDM does not stand in opposition to any of EBP's components. In contrast, it ensures that the application of scientific knowledge is meaningfully modulated by each patient's unique values, thereby fulfilling EBP's original promise as a paradigm that integrates science with humanism.

Without the formal recognition and practical enactment of SDM, the third pillar of EBP will remain a rhetorical abstraction, reducing patient autonomy to a merely legalistic formality devoid of substantive impact on clinical care. A coordinated effort is urgently needed across all levels of the healthcare system to elevate SDM to the status of a non‐negotiable element of EBP, thereby ensuring that patient autonomy transcends legal recognition and is realized as a lived experience within healthcare delivery.

## Conflicts of Interest

The author declares no conflicts of interest.

## Data Availability

Data sharing is not applicable to this article, as no new data were created or analyzed in this study.
